# Experimental Investigation of a Slip in High-Performance Steel-Concrete Small Box Girder with Different Combinations of Group Studs

**DOI:** 10.3390/ma12172781

**Published:** 2019-08-29

**Authors:** Bishnu Gupt Gautam, Yiqiang Xiang, Xiaohui Liao, Zheng Qiu, Shuhai Guo

**Affiliations:** 1 College of Civil Engineering and Architecture, Zhejiang University, Hangzhou 310058, China; 2 College of Civil Engineering and Architecture, Quzhou University, Quzhou 324000, China

**Keywords:** slip, group studs, composite beam, accelerated bridge construction, steel fiber

## Abstract

Due to the significant advantages of steel-concrete composite beams, they are widely used for accelerated bridge construction (ABC). However, there is still a lack of experimental research on the proper design of ABC, especially in the slip with a different group of shear connectors. As a component of steel-concrete composite structure, shear studs play a vital role in the performance of composite structures. This paper investigates the influence of group studs in simply supported and continuous box girders. To this end, three sets of simply supported steel-concrete composite small box girders and two continuous steel-concrete composite small box girders were made with different groups of shear studs, and the slip generated along the beams was recorded under different caseloads. The results were then compared with the proposed simplified equations. The results show that the slip value of the test beam is inversely proportional to the degree of shear connection. The slip of Simply Supported Prefabricated Beam-3 (SPB3) is 1.247 times more than Simply Supported Prefabricated Beam-1 (SPB1), and 2.023 times more than Simply Supported Prifabricated Beam-2 (SPB2). Also, the slip value of Experimental Continuous Beam-1 (ECB1) is 1.952 times more than Experimental Continuous Beam-2 (ECB2). The higher the degree of shear connection, the smaller the maximum slip value.

## 1. Introduction

With the development of urbanization and the rapid progress of civil engineering, the problem of urban congestion has become increasingly prominent. Therefore, it is necessary to improve the construction of urban transportation infrastructure methods and expand urban expressway networks. In order to build, replace, and repair a bridge, or a series of bridges, in areas with heavy traffic, traditional construction methods are usually used in current engineering projects. These methods generally include field activities, such as the installation of supports, formwork, binding of reinforcement bars, pouring and curing of concrete, etc. These onsite construction activities not only consume a great deal of time but also lead to traffic congestion, which weakens the safety and efficiency of the traffic network. On the other hand, due to the constraints of the construction site and climatic conditions, the components of onsite construction are prone to quality problems, which may potentially affect the durability of the structure. Accelerated bridge construction (ABC) is a promising approach to reduce the impact of construction on traffic, improve the quality of materials and durability of products, and minimize the construction time [1,2,3].

A combination of steel beams and a concrete deck with shear connectors, known as steel-concrete composite beams, are widely used in the ABC approach. Due to the use of shear stud tied together in the upper part of steel beam, this type of structure can give full strength to the respective properties of the two materials, especially for short and medium-span urban areas and highways [4,5,6,7]. The headed stud shear connectors are usually used to resist longitudinal slip and vertical separation between the steel beam and concrete slab. In general practice, construction of steel-concrete composite girder bridge, I-shaped steel beams, or a small steel box girder flange of the steel beam structure are used [8,9]. Compared with I-shaped steel girder, the small box section has better stability and torsional resistance. Moreover, compared with large box girders, small box girders have a flexible cross-section, lightweight, easy fabrication, and assembly, and thus, are more suitable for accelerated construction of the bridges [10].

The characteristics of concrete play a vital role in all kinds of structure for overall strength. Concrete is brittle and has partial ductile behavior. Therefore, a different form of reinforcement is needed to boost structural stability. Steel bars are used as reinforcement in concrete structures; however, there’s still a chance of crack, deflection and slip formation, which causes a big problem within the overall stability of the structure [11,12,13,14,15,16,17].

Concrete is a comparatively widely used material. However, it will run into huge issues and defects if it’s not prepared correctly. The defects are started from the concrete parts of reinforcement concrete members and extended until encountering a reinforcing bar in which yield to significant problems. We must control these defects to prolong the life period of concrete structures. Hence, according to the application of the concrete in the structure, a multi-directional and closely spaced reinforcement may need to be utilized. The use of fibers is another solution to enhance the ductility of concrete material. Fiber-reinforced concrete (FRC) consists of short distinct fibers that are uniformly distributed and at random bound among the concrete matrix. The fibers are mostly classified into four categories, namely—steel fiber, glass fiber, natural fiber, and synthetic fiber, all of which have their own respective properties [18,19,20,21,22].

The use of fibers in concrete components causes the increase of structural integrity, provides high tensile strength to plain concrete, decreases the permeableness of concrete, and increases the resistance to impact load. Fibers reduce the number of rebars without affecting the strength. It will additionally eliminate the defects by bridging action. Flexural behavior, bond strength, and particularly toughness of SFRC (steel fiber reinforced concrete) increases by increasing fiber content. Carbon or steel fibers are added to a cement matrix at a high volume fraction (0.5%–3%) to extend the conduction of the composite. The properties of fiber concrete depend on the quantity of fibers used [23,24,25,26,27,28].

Various problems are occurred in composite materials in terms of slip deflection and crack. The crack in a composite structure could decrease the stiffness and strength, which may accelerate the failure of the structure. Due to the inherent material properties of fiber concrete, the presence of fiber improves the resistance of conventionally reinforced structural members to serviceability deflection condition. Although the slip is not the main reason for a structural failure, it is part of the failure process due to losses in the structural integrity. Therefore, controlling slip plays an essential role in the failure mechanism of steel-concrete composite structures [29,30,31,32,33,34,35].

Generally, the concrete grade is below C50 in traditional construction, while high-performance concrete with a grade higher than C60 is used in the ABC process. Therefore, due to the high mechanical strength and durability, high-performance concrete in the composite beam receives much attention [36]. In the steel-concrete composite beams, there is a sliding problem in the interface layer between two materials, which is usually appeared by increasing load intensity. It can increase damping, which is beneficial for dynamic loading conditions [37,38]. The existence of such an interface slip can reduce the stiffness, increase the curvature and the deformation of composite beams [39,40,41,42,43,44,45]. Therefore, the actual working condition of composite beams cannot be precisely determined without considering the interface slip effect. In the past few years, many studies attempted to address the slip problem; however, few of them, if any, considered the group studs in the beam [46,47,48,49,50,51,52]. So, this research is only focused on the slip tests of a steel-concrete composite small box girder (SCCSBG) in the simply supported and continuous beams.

In this research, two equations were proposed, one for the simply supported beam and another for continuous beams to consider the influence of group studs and compare their performance. For this purpose, three simply supported SCCSBG (steel-concrete composite small box girder), and two continuous SCCSBG were experimentally tested under the action of the vertically applied load. The slip values were observed throughout the full span of the beam. The details of the experiments and their behavior are discussed in the following sections.

## 2. Design and Fabrication of Experimental Beams

The experimental SCCSBG beams, which were made using accelerated construction techniques, consist of an open steel box girder, diaphragm, welded stud, and concrete slab. Due to difficulties in the process of making a real-size scale model, especially in the case of the concrete bridge deck and the thickness of the steel plate, it followed the design specification. Based on the 25 m span prototype bridge, a stereotype model of steel concrete steel fibrous small box girder was prepared to the actual length ratio of 1:4 and 1:6 for continuous and simply supported beams, respectively. The ECB-1 and ECB-2 were used for the continuous beam, whereas SPB1 to SPB3 were used for the simply supported beam. The material details about both types of the beams are listed in Table 1.

In the accelerated construction of SCCSBG, the spacing of studs, group arrangement of studs, and concrete materials are the three main factors. Using the high-performance concrete reduces the structural weight and cost and increases the durability. Therefore, recently, it is more widely used in bridge engineering. The spacing and arrangement of the studs should be determined using the degree of shear connection. The degree of shear connection is the ratio of the number of actual welded studs in the shear span to the number of welded studs required for the complete shear connection. In the designing procedures, while considering an arrangement for studs, a minimum space according to the degree of shear connection is defined to ensure the mechanical performance of the structure. However, in term of construction, having more space between the group studs is preferred. To design the test beams, the degree of the shear connection was determined based on the designing of steel and concrete composite bridges code GB50917-2013 [53]. However, there is no code available to specify the arrangement and spacing of group studs. Therefore, the formula of bearing capacity for a single stud defined in this code is used here. EC4 [54] requires that the maximum spacing of stud uniformly distributed along the steel-concrete composite beams should not exceed four times of the concrete slab thickness or the value of 800 mm.

Based on the steel structure design code GB50017-2017 [55], if the strength and deformation are satisfied, the longitudinal shear capacity of the shear connectors at the interface of composite beams guarantees the full flexural capacity of the beam. Hence, the beam can be designed according to the partial shear connection. However, the partial shear connection is limited to the composite beams with equal cross-section and the spans less than 20 m. The American Association of State Highway and Transportation Officials (AASHTO) [56] bridge design code stipulates that the spacing of reserved holes is not more than 610 mm.

**Figure 1:**
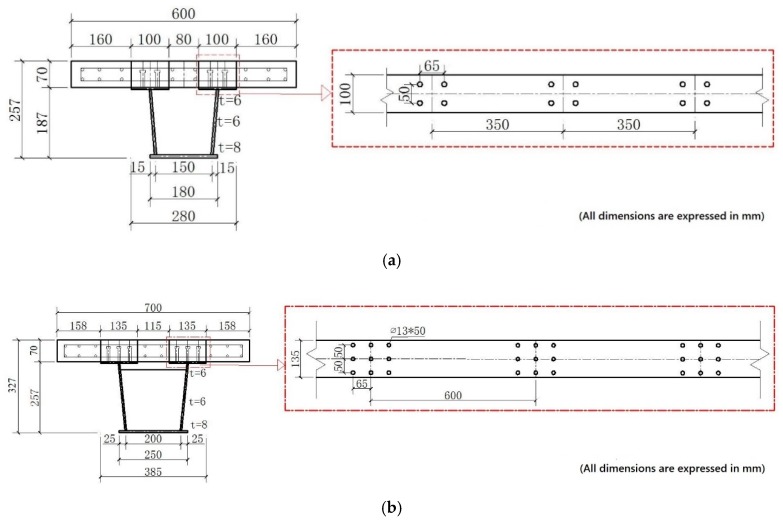
Details of stud arrangement cross-section of experimental accelerated construction steel-concrete steel fibrous high-performance composite box girder bridge. (**a**)Typical arrangement of a simply supported beam (SPB1); (**b**) Typical arrangement of a continuous beam (ECB-2).

In the fabrication, two different lengths of girders are used to manufacture the beams. The smaller one is used in the SPBs, and the larger one is used in the ECBs. The SPBs are 4.5 m in length, 4.2 m in support spacing, 258 mm in depth of the composite beam and 70 mm in the thickness of the precast concrete slab. For the steel U-type girder, the depth is 0.187 m, the top width is 180 mm, and the bottom width is 150 mm. The upper flange is 6 mm thick and 100 mm wide. The web is 6 mm thick, and the bottom is 8 mm thick. A solid web diaphragm is set every 700 mm from support. The diaphragm is 6 mm thick and 160 mm high. The steel U-type girder is made of Q345qc steel. The group studs are welded to the upper flange of the steel beam. The dimension and materials of welding for studs are 13 mm × 45 mm and ML15AL, respectively. The SPBs were made of C60 high-performance concrete with a width of 600 mm and a thickness of 70 mm. The center distance of the group studs varies from 350 mm to 420 mm from support to midspan. Two layers of steel bars were arranged longitudinally. The upper layer of steel bars is HRB400 with a diameter of 10 mm, and the lower layer of steel bars and stirrups are HPB300 with a diameter of 8 mm. The spacing of stirrups is 60 mm, and no stirrups are arranged in the reserved hole position. The details about stud arrangement and cross-section of the SPBs is shown in Figure 1a. The design parameters of SPBs and ECBs are presented in Table 2.

**Figure 2:**
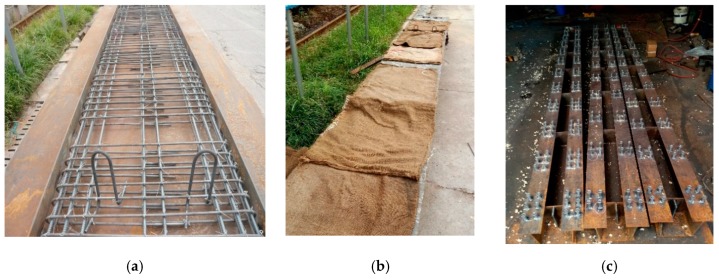

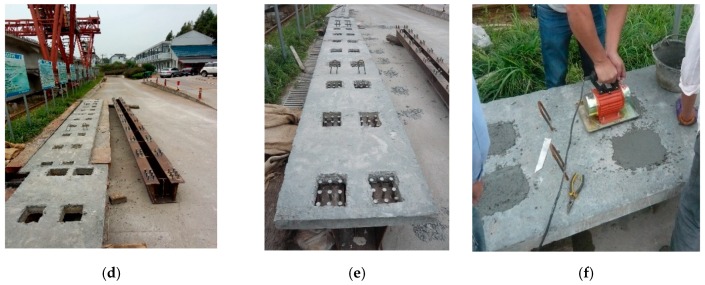
Fabrication process of the experimental beams: (**a**) formwork erection and reinforcing case binding; (**b**) casting and curing of concrete slab; (**c**) stud welding of test specimens; (**d**) completion of the concrete slab and steel U-type girder; (**e**) placing of concrete slab on steel box girder to make it composite; (**f**) reserve hole filling.

ECBs were made of two spans, each span is 3 m in length, with a total length of 6.3 m long. The total depth of the composite box girder was 327 mm with 70 mm slab thickness and 257 mm depth of steel U-type girders. The upper flange was 6 mm thick and 135 mm wide. The essential of the web was 6 mm, whereas the bottom plate was 8 mm. The diaphragm was set to every 600 mm from support to throughout the box girder. The thickness and height of the diaphragm were 6 mm and 220 mm, respectively. The construction of prefabricated concrete slabs, welding steel beams, and welding studs was in accordance with the requirements of design drawings and technical construction specifications. The C60 high-performance concrete and C80 steel fiber high-performance concrete are used for prefabricated slab and reserved hole filling, respectively. The detail configuration of both types of box girder is shown in Figure 1, and the main fabrication process is shown in Figure 2.

For the high-performance concrete element, copper-plated steel fiber with a diameter of 0.2 mm and length of 13 mm, tensile strength of 2000 MPa, and a volume fraction of 1.5% was used. The details about the physical properties of the steel fiber are listed in Table 3. The obtained physical properties by the use of cement, fine aggregate, coarse aggregate, water, and chemicals are based on different codes [57,58,59]. The information about different of properties and materials uses are described in Appendix A. The arrangement of bars in the ECB’s are the same as SPB’s. However, for the stud arrangement, the hole with a plane size of 160 mm × 100 mm is reserved and filled with C80 steel fiber concrete. The size of the ECB’s welding stud is 13 mm × 50 mm.

## 3. Experimental Test

### 3.1. Test Method and Instrumentation

**Figure 3:**
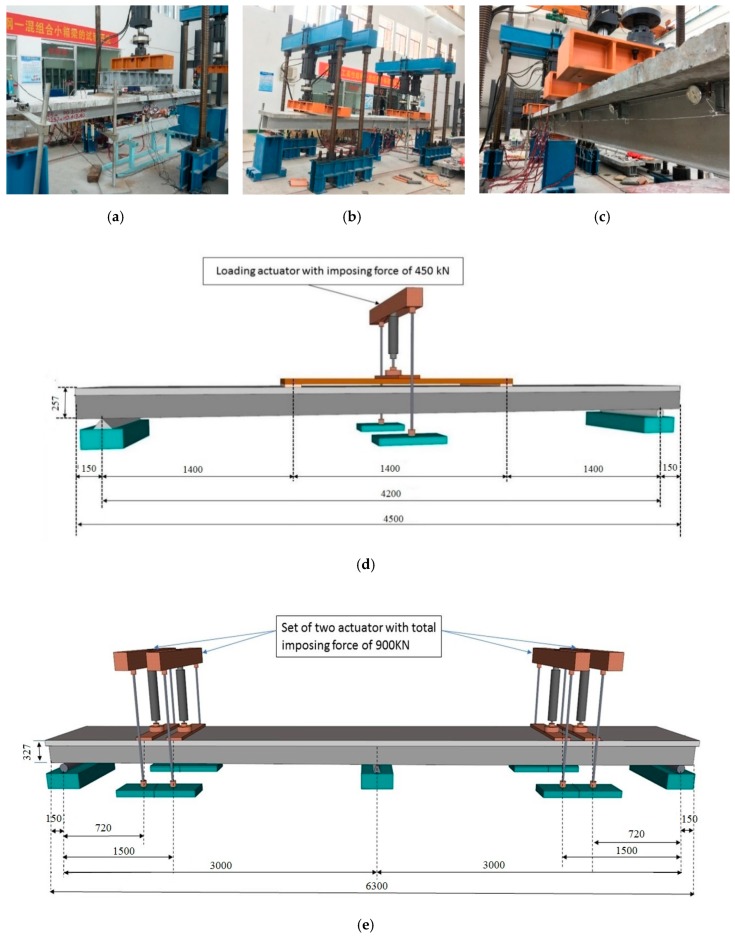
Experimental setup for slip measurements (m); (**a**) Loading system of SPBs (**b**) Loading system of ECBs; (**c**) Arrangement of dial gauges; (**d**) Schematic view of SPBs (mm); (**e**) Schematic view of ECBs (mm).

The relative slip between the concrete, slab, and steel U-type girder was measured by means of the dial gauge (Wanmu, Chengdu, China). The dial gauges were fixed at the lower edge of the concrete slab, and free end contacts with the aluminum block extending from the bottom of the upper flange of the steel U-type girder. The dial gauges were installed along the experimental beams under the group studs, as shown in Figure 3c. Each beam is placed with a dial gauge at the position of group studs from the support to the throughout the span. Due to have a symmetric shape, the dial gauges are mainly arranged in only one side.

### 3.2. Loading Procedure

The 500 kN servo loading system (Popwil, Hangzhou, China was used for the test loading. The boundary conditions of the beams were roller and hinge bearing at both ends of the beam. The experiment was performed in the structural lab of Quzhou University in China. The loading was directly exerted to an area of 60 cm × 20 cm of the experimental beam. For SPBs, the loading was symmetrically applied at two areas within the full span. The distance between the loading areas is 1.4 m. A layer of fine sand is cushioned at the loading area to ensure that the load of the test beam is uniformly applied to the beam. The loading, as mentioned above procedure, was performed for SPBs. Whereas, for the ECBs, a set of two actuators with the total loading capacity of 1000 kN was employed. The loading was applied at each mid-span of a two-span continuous SCCSBG with a loading area of 70 cm × 20 cm. The ECB’s were supported by the roller bearing at both ends and a hinge bearing at mid support. The loading increment applied in the laboratory model was about 5% of the yield load (Py) in each load step. The slip values of test beams were carefully measured throughout the beams, which is shown in Figure 3c. The loading arrangement of the test beams are shown in Figure 3d,e. The full experimental detail is shown in Figure 3. Before the formal loading, two preloads were carried out to verify the normal working of test instruments and meet the quality test requirements.

### 3.3. Slip Measurement

**Figure 4:**
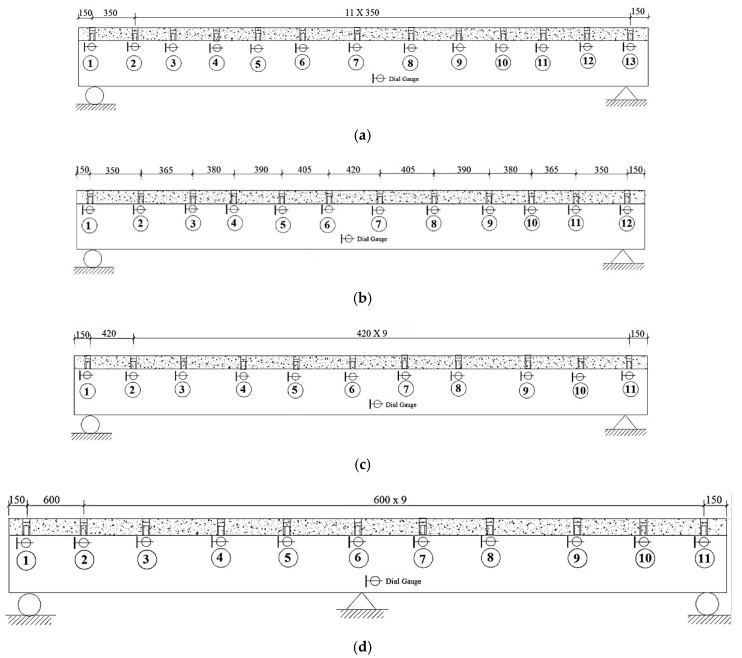
Location of dial gauges (mm); (**a**) SPB1; (**b**) SPB2; (**c**) SPB3; (**d**) ECB-1 and ECB-2.

**Figure 5:**
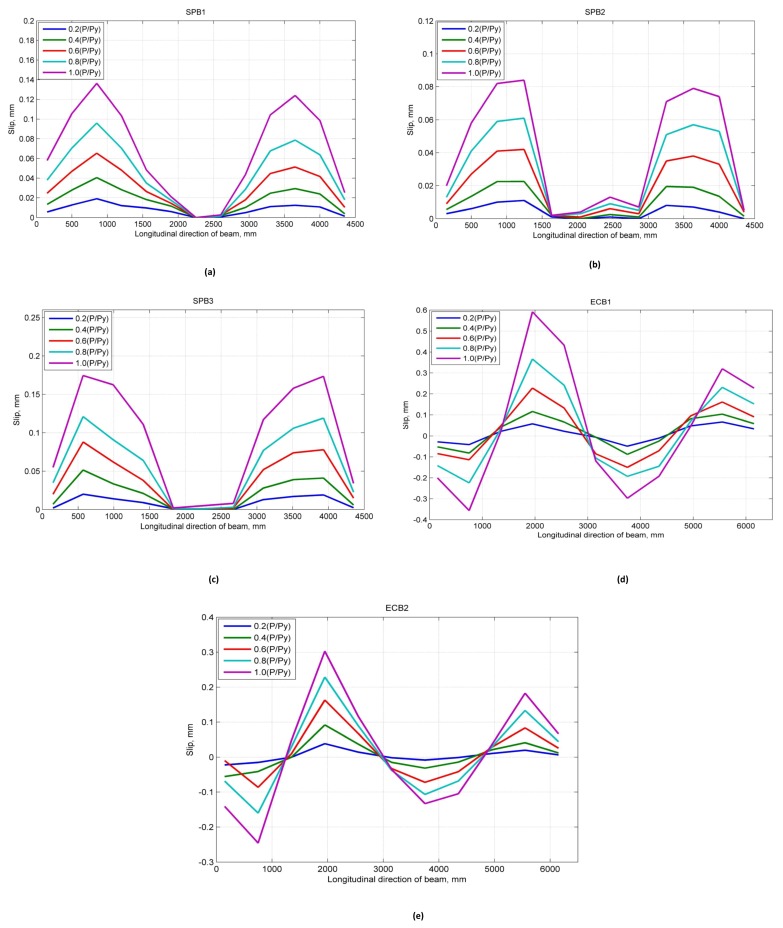
Variation of measured interface slip with a longitudinal direction of the beam under the action of vertical loads: (**a**) SPB1; (**b**) SPB2; (**c**) SPB3; (**d**) ECB1; (**e**) ECB2.

The slip mainly occurs in the shear span between support and the loading point. The slip values in the pure bending segment of the beams and the midspan are minimal. There is no shear force at the section of the steel beam and concrete slab in the pure bending segment in the middle of the experimental beam. In the general concept, there should be no slip, but as the deflection of the beam increases, the concrete slab near the loading point has an oblique extrusion effect on the welding studs, which makes the relative displacement between the steel beam and a concrete slab in a longitudinal direction. Because of the boundary condition, the maximum slip value does not appear in the support region, but it occurs at the shear span segment. Moreover, because of the different restrained strength of the support at both ends, the slip of the supports is asymmetric. The arrangement of dial gauges to measure slip values are shown in Figure 4. The experimental slip results in the longitudinal direction of the SPBs and ECBs under the action of vertical load are shown in Figure 5.

## 4. Simplified Model

The slip was observed under 24 kN of load where the values of SPB1, SPB2, and SPB3 were 0.009, 0.004 and 0.008 mm, respectively. The slip values of the SPBs increases with the increase of the load. As shown in Figure 5, the relationship between the growth rate of the SPBs slip values are SPB3> SPB1 > SPB2, and it does not increase linearly with the increase of the load. The inconsistency growth rates of slip values are mainly affected by the degree of shear connection. Therefore, as the load increases, the welding studs gradually entered into yielding. So, the growth rate of slip values increases continuously, and the slip value of each stud group in the shear span tends to be uniform.

The maximum slip values of SPB1, SPB2, and SPB3 under 270 kN load are 0.136, 0.084, and 0.170 mm, respectively. The maximum slip values have an inverse relationship with the degree of shear connection. The larger the degree of shear connection, the smaller the slip value. The main reason for this is the local effect of group studs shear connectors. The location of reserved holes is being centralized, and the shear stiffness of this location is becoming more significant than the traditional uniformly distributed studs.

There are so many conditions which need an ideal mathematical solution to represent a set of data. Using the N-order polynomial regression model, a line can be fitted to the experimental scatter data, and formula can be extracted to represent/estimate the data [60]. Therefore, two quadratic equations extracted using N-order polynomial regression are proposed in the form of Equations (1) and (2). The residual value for each data point is the distance from the target point to the regression line, which is error in prediction. The norm of residual is a measure of goodness of fit, so that a value centered around zero indicates a better fit [61]. Equation (1) is for SPBs, while Equation (2) addresses ECBs. The norm of residuals for Equations (1) and (2) are 0.027 and 0.112, respectively.

P/Py = −14 × s^2^ + 8.1 × s + 0.014 (1)

**Figure 6:**
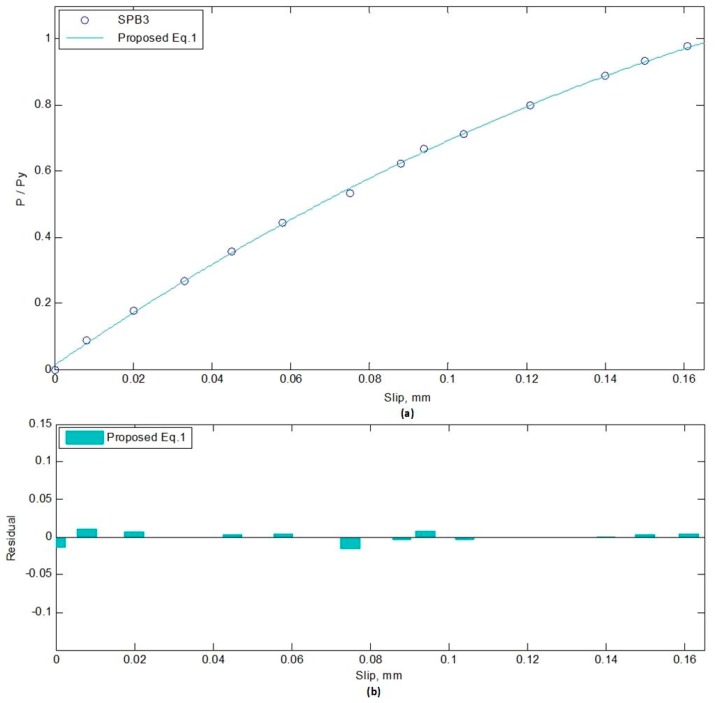
Fitting of curve, SPB3: (**a**) Load-slip; (**b**) Residual-slip.

**Figure 7:**
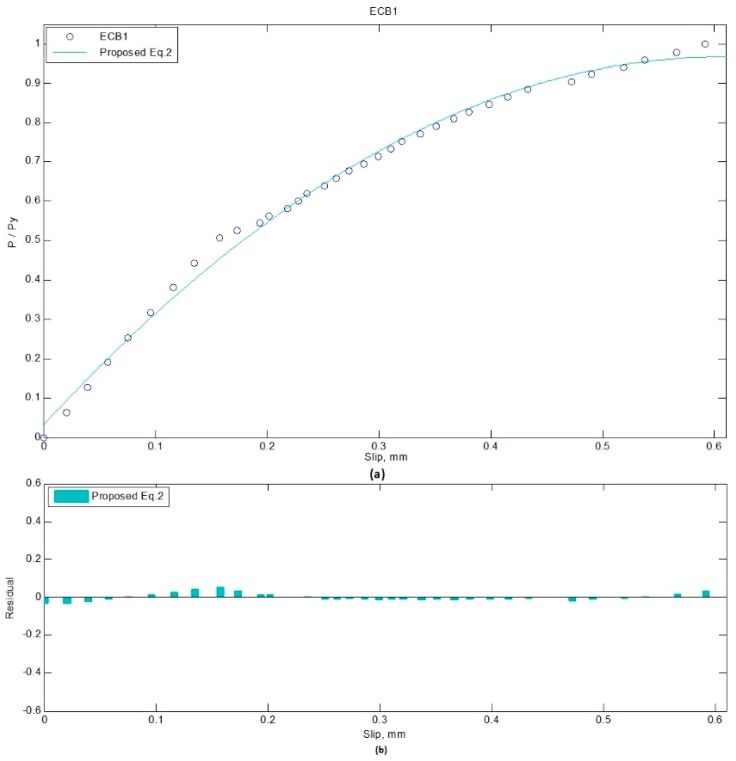
Fitting of curve, ECB1: (**a**) Load-slip; (**b**) Residual-slip.

P/Py = −2.5 × s^2^ + 3.1 × s + 0.031 (2)

where P/Py is the ratio of an applied load to yield load, and s is the slip of the beam at corresponding load. The proposed equation can be used for the prediction of the slip in SCCSBG of simply supported and continuous beam with fully composite action. When the degree of shear connection is equal, these equations can predict the slip value of that beam. Whereas, with a different degree of shear connection, it can relate the change in slip values between them. However, the limitation of the proposed equation regarding the dimension and construction size should also be considered while applying it. Figure 6 and Figure 7 show the proposed equations with the experimental data and corresponding residual values. The residual (%) in the fitting process of SPB3 and ECB1 load-slip curves are presented in Table 4 and Table 5, respectively.

## 5. Discussion

**Figure 8:**
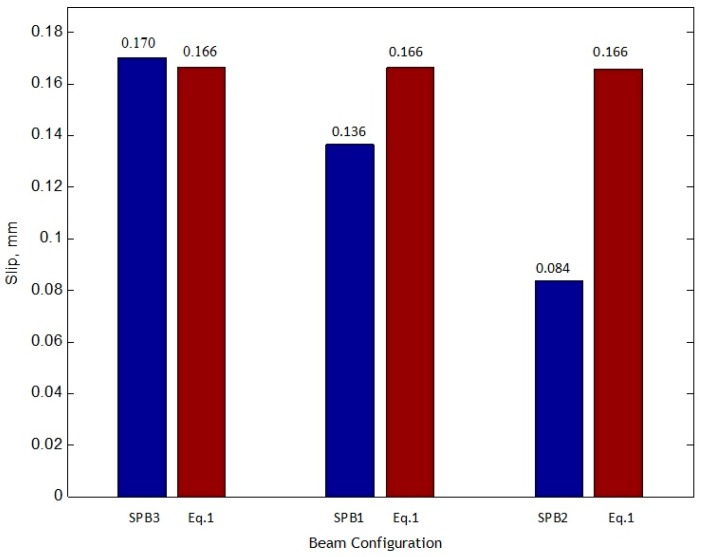
Comparison of proposed Equation (1) with SPB1, SPB2, and SPB3.

**Figure 9:**
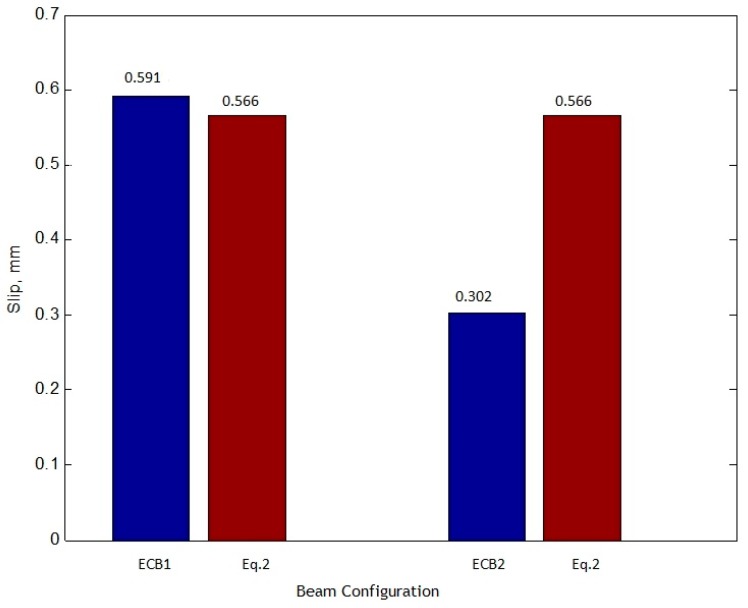
Comparison of proposed Equation (2) with ECB1 and ECB2.

Based on the experimental results, the relationship between applied load and interface slip was studied, and it is noteworthy that all the provided relationships are limited to the upper yield (Py) point of the elastic stage. The upper yield load is around 270 kN for the SPBs and 900 kN for the ECBs. To examine the characteristics performance of the different types of group studs by using the proposed equations, the comparison between all the SPBs and ECBs were performed. The comparison of the proposed equations and experimental results of the SPBs are presented in Table 4. The same comparison for ECB’s are also reported in Table 5. With regard to the different group studs arrangement, by increasing the intensity of the load ratio (P/Py), the smaller group size with a more number of welding studs has a lower value for a slip and vice versa. The diagram of the measured yield load (Py) to the corresponding slip for SPBs and ECBs are illustrated in Figure 8 and Figure 9, respectively. The higher degree of shear connection for simply supported beam in the SPB2 and for continuous beam in ECB2 causes less slip value. The proposed Equation (1) and SPB3 have an almost similar value of slip, so it is considered as Equation (1) with a degree of connection similar to SPB3. Equation (2) also has a degree of shear connection similar to ECB1. The slip value of SPB3 is 1.250 times more than SPB1, and 2.023 times more than SPB2. Also, the slip value of ECB1 is 1.952 times more than ECB2.

## 6. Conclusions

This study focused on the static behavior of the accelerated construction steel fibrous high-performance steel-concrete composite small box girder bridge. To achieve this, two different types of simply supported and continuous beams have been tested with trapezoidal steel beams and different shear stud arrangements. Each sample was tested under a progressive applied vertical loading, and the maximum slip was recorded at key points along the span for both simply supported and continuous beams. Using the dataset obtained from laboratory tests, simplified empirical relationships were developed to approximate the slip of both simply supported and continuous beams with a different degree of shear connections.

The following main conclusions are drawn according to investigation in this paper.

- It was observed that the measured slip values for SPB1, SPB2, and SPB3 samples of simply supported beams at 0.2 (P/Py) were 0.019 mm, 0.011 mm, and 0.020 mm, respectively. While the slip values for ECB-1 and ECB-2 samples of continuous beams were 0.057 mm and 0.038 mm, respectively.
- The integrity and load-carrying capacity of accelerated construction high-performance SCCSBG are found to be more than traditional construction because of the use of steel fibers in concrete. Therefore, for SPB1, SPB2 and SPB3, the slip values of simply supported beams at 1.0 (P/Py) are bounded to 0.136 mm, 0.084 mm and 0.174 mm, respectively. In addition, for continuous beams, the sliding values measured by ECB-1 and ECB-2 at 1.0 (P/Py) are limited to 0.591 mm and 0.303 mm, respectively.
- Because of the higher degree of shear connection, the slip of SPB3 is 1.247 times more than SPB1, and 2.023 times more than SPB2. Also, the slip value of ECB1 is 1.952 times greater than ECB2. That is, the higher the degree of shear connection is, the smaller the maximum slip value becomes.

Since the proposed equations are presented in an explicit form, they can be practically used to predict the slip of group studs in either simply supported or continuous beam. As the experimental model was designed with ratios of 1:6 and 1:4 for simply supported and continuous beams, respectively, the application of the proposed model is limited to cases with a similar range of parameters. Other sizes and forms of accelerated construction beams can be investigated by the authors in future research.

## Figures and Tables

**Table 1:** Material details of experimental beams.

Material	Parameter	Value
Concrete	Density, (kg/m^3^)	2400
	Elastic modulus, (MPa)	36,400
	Poisson’s ratio	0.167
	Design strength of bridge deck concrete, (MPa)	C60
	Reserved hole concrete design strength, (MPa)	C80
	Yield strength, (MPa)	76.24
Steel (Q345qc)	Density, (kg/m^3^)	7850
	Elastic modulus, (GPa)	210
	Poisson’s ratio	0.3
	Yield strength, (MPa)	421
steel bar (HPB 300 and HRB 400)	Density, (kg/m^3^)	7800
	Elastic modulus, (GPa)	206
	Poisson’s ratio	0.3
	Yield strength, HRB400, (MPa)	445
	Yield strength, HPB300,(MPa)	363
Stud	Density, (kg/m^3^)	7800
	Elastic modulus, (GPa)	210
	Poisson’s ratio	0.3
	Yield strength, (MPa)	360

**Table 2:** The design parameters of SPBs and ECBs.

Parameter	SPB1	SPB2	SPB3	ECB1	ECB2
Size of stud (mm)	Φ13 × 45	Φ13 × 45	Φ13 × 55	Φ13 × 50	Φ13 × 50
Distribution of studs (Tr. × L.)	2 × 2	2 × 2	2 × 3	2 × 3	3 × 3
Longitudinal spacing between adjacent group of studs (mm)	350	350–420	420	600	600
Total number of studs	104	144	88	132	198
Degree of shear connection	1.13	1.57	0.96	1.02	1.53
Transverse c/c spacing of studs	50	50	50	50	50
Longitudinal c/c spacing of studs	65	65	65	65	65
Beam total length (m)	4.5	4.5	4.5	6.3	6.3
Combined beam width (mm)	600	600	600	700	700
Beam height (mm)	257	257	257	327	327
Thickness of concrete deck (mm)	70	70	70	70	70

**Table 3:** Physical properties of steel fibers.

Fiber Type	Length (mm)	Diameter (mm)	Aspect Ratio	Tensile Strength (Mpa)	Modulus of Elasticity (GPa)	Density (Kg/m^3^)
Steel fiber	13	0.2	65	2000	210	7800

**Table 4:** Comparison of slip between the test results of SPBs and the proposed equation.

Measured P/Py	Slip, mm				Residual (%) SPB3
	SPB1	SPB2	SPB3	Proposed Equation (1)	
0.2	0.019	0.011	0.020	0.020	0
0.4	0.040	0.022	0.051	0.051	1.360
0.6	0.065	0.042	0.088	0.088	0.113
0.8	0.096	0.061	0.121	0.122	1.150
1.0	0.136	0.084	0.170	0.166	2.264

**Table 5:** Comparison of slip between the test results of ECBs and the proposed equation.

Measured P/Py	Slip, mm			Residual (%) ECB-1
	ECB-1	ECB-2	Proposed Equation (2)	
0.2	0.057	0.038	0.045	22.393
0.4	0.116	0.092	0.103	10.966
0.6	0.228	0.163	0.221	3.027
0.8	0.367	0.229	0.378	3.217
1.0	0.591	0.303	0.566	4.226

**Figure A1:**
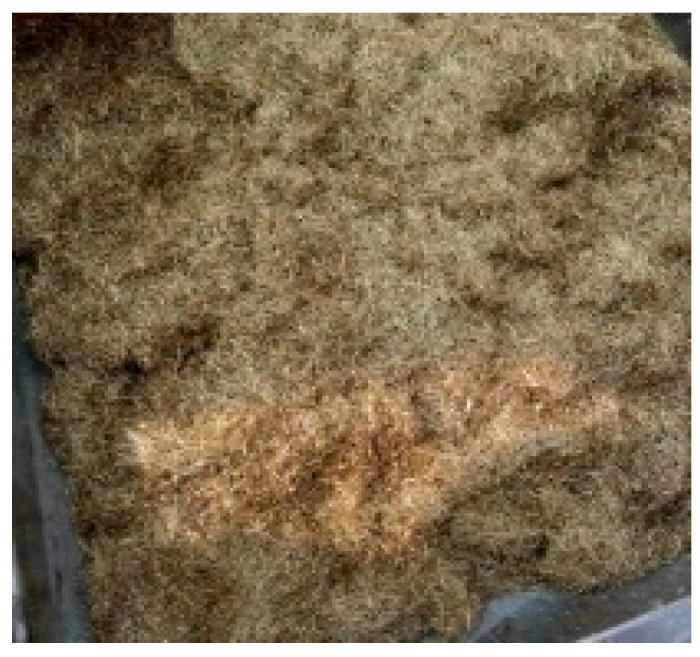
Steel fiber in concrete.

## Appendix A

A local brand cement of 52.5 grade OPC of and medium sand from Ganjiang river was used for fine aggregate with a fineness modulus of 2.8. For coarse aggregate, well-graded basalt was used with a maximum particle size less than 16 mm. The main chemical composition for bonding properties of the constituent materials are shown in Table A1. The average fineness of the slag powder, density, water reduction rate, activity index on 28d, and activity index on 56d were 3 microns, 2.40 g/cm^3^, 10–15%, 105–110% on 28d, and 110–125%, respectively.

**Table A1:** Chemical constituents of Diatomite, SiO_2_ and mineral powder (%).

Component	SiO_2_	Al_2_O_3_	MgO	CaO	f-CaO	SO_3_	MnO	Density	Loss
Diatomite	76.11	11.21	3.5	3.8	-	-	-	2.6	<1
Sub-nano SiO_2_	80.11	1.49	2.69	4.64	1.98	0.65	-	-	<1.5
Micron grade Mineral powder	33.84	11.68	10.61	38.13	-	-	0.34	-	<3

Silicon Powder was made by local Building Materials Co., Ltd. according to GB/T176-1996 [56]. The test results of Silicon powder measured by GB/T18736/2002 [57] is reported in Table A2.

**Table A2:** Silicon powder performance index.

Item	SiO_2_ (%)	Moisture Content (%)	Ignition Loss (%)	Water Demand Ratio (%)	Fineness (45 um) (%)	28d Activity Index
Control index	≥85	≤3.0	≤6	≤125	-	≥85
Test Result	95.3	0.90	2.1	119.5	0.9	90

Steel fibers: Straight copper-plated steel fibers with a length of 13 mm, the diameter of 0.2 mm, the volume fraction of 1.5%, the tensile strength of 2000 MPa, and were used as steel fibers. Figure A1 shows the steel fibers mixed with C80 high-performance concrete.

Water reducer: Superplasticizer of a local chemical company was used. The index parameters are listed in Table A3.

**Table A3:** Performance index of water reducer.

Item	PH Value	Density (g/mL)	Solid Content (%)	Chloride Ion Content (%)	Alkali Content (%)	1 h Loss	Water Reduction Rate.
Control Index	6.5 ± 0.02	1.04 ± 0.02	21 ± 1	≤0.2	≤3.0	40	≥25
Test result	6.5	1.046	21.5	0.05	0.12	23	30.4

Water: Ordinary tap water was used in the experiment.

Each concrete was designed with three mixing ratios and after that, C60 concrete, and C80 concrete was tested. Three sets of test pieces were made for each combination, and each group had three cube test pieces. After conducting the strength test, the design mix ratio was optimized. The concrete mix of C60 and C80 is shown in Table A4.

**Table A4:** Benchmark mixture proportion of C60 and C80 high-performance concrete.

Concrete	Water Binder Ratio	Sand Rate (%)	The Amount of Raw Material Used per Concrete							
			Cement	Slag Powder	Fine Aggregate	Coarse Aggregate	Water	Water Reducer	Silica Fume	Steel Fiber
C60 *	0.3	40	388	129	699	1049	155	6.2	-	-
C80 **	0.25	38	435	87	652	1063	157	10.44	58	87

* Cement = 0.75, slag powder = 0.25, fine aggregate = 1.35, coarse aggregate = 2.03, water = 0.3, Admixture = 0.012. ** Cement = 0.75, slag powder = 0.15, Silica powder = 0.1, fine aggregate = 1.124, coarse aggregate = 1.83, steel fiber = 0.15, water = 0.27, and water reducer = 0.018.

C60 and C80 high-performance concrete were used in the test of the beam. For C60, eight groups of 150 mm cubic test specimen and two groups of 150 mm × 150 mm × 300 mm prism specimen were made. Four groups of cube specimen were tested with the same curing condition as of beam. Where, with two groups of the cube, compressive strength was measured with a temperature of 600 °C. One group of the specimen was tested on the day of testing of the beam, and other was preserved. The four groups which remained was used for standardization; among them, two groups and one group were used separately for cube test of 7 days and 28 days compressive strength respectively, and last one group was used for testing splitting strength. C80 steel fiber high-performance concrete was used to pour in the reserved hole, and at the same time, three cubic specimen and two groups of prism were made. Among the cubic specimen, two groups were used to obtain 28-days cubic strength and splitting strength, and the remaining one group was preserved. Simultaneously, the remaining two groups of prism specimens were used to test 28-day compressive strength and elastic modulus of concrete. This both types, cubic and prismatic specimens were tested in a universal testing machine. The test results and elastic modulus of C60 high-performance concrete cube under standard curing and same curing condition with tested beam are shown in Table A5, Table A6 and Table A7. The test results of C80 steel fiber high-performance concrete cube and elastic modulus are shown in Table A8 and Table A9.

**Table A5:** Concrete strength obtained from the experimental test on standard C60 HPC cubic specimen.

Test Block Number	Standardized 7-Day Cube Strength (MPa)		Standardize 28-Day Cube Strength (MPa)	Splitting Strength (MPa)
Test block 1	63.4	55.9	77.8	5.63
Test block 2	55.2	54.1	69.9	5.08
Test block 3	48.9	57.9	67.2	4.56
Average value	55.9		71.6	5.09

**Table A6:** Concrete strength obtained from experimental test on C60 HPC cubic specimen at the same condition with bridge deck.

Test Block Number	Compressive Strength (MPa) *		Strength Measured at the Test Day (MPa)
Test block 1	71.3	64.9	77.87
Test block 2	71.4	61.3	75.37
Test block 3	64.7	69.3	75.47
Average value	67.15		76.24

***** Strength measured at the same curing condition with bridge deck.

**Table A7:** Elastic modulus obtained from the experimental test on C60 HPC.

Item	First Group	Second Group	Third Group	Average Value
Modulus of Elasticity (GPa)	36.0	36.7	36.5	36.4

**Table A8:** Concrete strength obtained from the experimental test on standard C80 HPC cubic specimen.

Test Block Number	Standard Curing 28-Day Strength (MPa)	Splitting Strength (MPa)
Test block 1	90.4	8.27
Test block 2	87.5	9.91
Test block 3	84.4	8.83
Average value	87.4	9

**Table A9:** Elastic modulus obtained from the experimental test on C80 HPC.

Item	First Group	Second Group	Third Group	Average Value
Modulus of elasticity (GPa)	37.1	36.1	36.3	36.5
